# The effectiveness of non-surgical interventions for common plantar digital compressive neuropathy (Morton’s neuroma): a systematic review and meta-analysis

**DOI:** 10.1186/s13047-019-0320-7

**Published:** 2019-02-13

**Authors:** Barry G. Matthews, Sheree E. Hurn, Michael P. Harding, Rachel A. Henry, Robert S. Ware

**Affiliations:** 1 School of Clinical Sciences, Queensland University of Technology, Kelvin Grove, Brisbane, QLD 4059 Australia; 2 Institute of Health and Biomedical Innovation, Queensland University of Technology, Kelvin Grove, Brisbane, QLD 4059 Australia; 3 School of Health Sciences, University of South Australia, Adelaide, SA 5000 Australia; 4 Rachel Henry Podiatry, Clayfield, Brisbane, QLD 4011 Australia; 5 Menzies Health Institute Queensland, Griffith University, Nathan, Brisbane, QLD 4111 Australia

**Keywords:** Common plantar digital nerve, Morton’s neuroma, Compression neuropathy, Non-surgical intervention

## Abstract

**Background:**

Morton’s neuroma (MN) is a compressive neuropathy of the common plantar digital nerve. It is a common compressive neuropathy often causing significant pain which limits footwear choices and weight bearing activities. This paper aims to review non-surgical interventions for MN, to evaluate the evidence base for the clinical management of MN.

**Methods:**

Electronic biomedical databases (CINAHL, EMBASE, MEDLINE and Cochrane) were searched to January 2018 for studies evaluating the effectiveness of non-surgical interventions for Morton’s neuroma. Outcome measures of interest were treatment success rate (SR) (binary) and pain as measured using 100-point visual analogue scale (VAS) (continuous). Studies with and without control groups were included and were evaluated for methodological quality using the Downs and Black Quality Index. Results from randomised controlled trials (RCT) were compared between-groups, and case series were compared pre- versus post-treatment. Effect estimates are presented as odds ratios (OR) for binary data or mean differences (MD) for continuous data. Random effects models were used to pool effect estimates across studies where similar treatments were used. Heterogeneity was assessed using the *I*^*2*^ statistic.

**Results:**

A total of 25 studies met the inclusion criteria, seven RCTs and 18 pre/post case series. Eight different interventions were identified, with corticosteroid or sclerosing injections being the most often reported (seven studies each). Results from a meta-analysis of two RCTs found corticosteroid injection decreased pain more than control on VAS (WMD: -5.3, 95%CI: -7.5 to − 3.2). Other RCTs reported efficacy of: manipulation/mobilisation versus control (MD: -15.3, 95%CI: -29.6 to − 1.0); extracorporeal shockwave therapy versus control (MD: -5.9, 95%CI: -21.9 to 10.1). Treatment success was assessed for extracorporeal shockwave therapy versus control (OR: 0.3, 95%CI: 0.0 to 7.1); and corticosteroid injection vs footwear/padding (OR: 6.0, 95%CI: 1.9 to 19.2). Sclerosing and Botox injections, radiofrequency ablation and cryoneurolysis have been investigated by case series studies, however these were of limited methodological quality.

**Conclusions:**

Corticosteroid injections and manipulation/mobilisation are the two interventions with the strongest evidence for pain reduction, however high-quality evidence for a gold standard intervention was not found. Although the evidence base is expanding, further high quality RCTs are needed.

**Electronic supplementary material:**

The online version of this article (10.1186/s13047-019-0320-7) contains supplementary material, which is available to authorized users.

## Background

Morton’s neuroma (MN) is a compressive neuropathy of the common plantar digital nerve, most commonly occurring in the third web space, followed by the second and then the fourth [1–10]. The plantar nerve enlargement was first described in 1835 [11], the symptoms in 1845 [12] and the condition was initially called metatarsalgia in 1876 by Thomas Morton whose name is now associated with the condition [13]. Affecting 88 in every 100,000 women and 50 in every 100,000 men presenting for primary care in the United Kingdom, it is the most common compressive neuropathy after carpal tunnel syndrome [14].

People with MN usually describe abnormal forefoot sensations such as a burning or ache [15]. Pain localisation is most common in the plantar aspect of the forefoot, followed by the toe(s) and then the dorsal web space [16]. Diagnosis can reliably be made based on clinical presentation and testing [15] with ultrasound proposed as an accurate and cost-effective imaging method to confirm the diagnosis, especially in cases where the clinical diagnosis is equivocal [17]. Ultrasound without a clinical diagnosis may lead to a false diagnosis of MN for asymptomatic interdigital nerve enlargements [18].

Confusion surrounds the name MN, with many alternative descriptions for the condition (e.g. intermetatarsal neuroma [5, 19], intermetatarsal neuritis [5], plantar interdigital neuroma [1, 7, 20], interdigital neuroma [3, 4, 10, 21], interdigital neuralgia [22], interdigital neuritis [23] and plantar digital neuralgia [2]) and no histological evidence of a true neuroma with axonal degeneration and collagen proliferation [24].

Non-surgical interventions for MN are a recommended treatment option before surgery [25–28]. Clinicians also encounter health consumers who either decline surgical intervention or, due to contraindications, are not suitable for surgery. A comprehensive review of the non-surgical interventions would benefit all clinicians managing this cohort. Clinicians may recommend a range of treatments for MN. Current published treatment pathways for the non-surgical management of MN are based on a combination of one RCT [3], a number of pre/post case series, and expert opinion. These pathways follow a staged care approach from wider, low heeled footwear and metatarsal padding [25–28], foot orthoses [26, 29] or oral non-steroid or steroid medications [26] to corticosteroid injections [25–30], sclerosing injections [25–28] or extracorporeal shockwave therapy (ESWT) [26] and end with surgical interventions [25–30] including cryoneurolysis [25, 26]. However, at present there is no clarity surrounding which treatment options are most effective and no standard practice around which treatment should be considered the gold standard.

A 2004 Cochrane review that assessed interventions for MN included three randomised controlled trials (RCT), with two surgical and one non-surgical intervention [31]. The review concluded there was insufficient evidence to assess the effectiveness of interventions for MN. Another systematic review, based on a 2015 search, concluded that the effectiveness of non-surgical treatments appears less than surgical but “the paucity of adequate studies makes it hard to assess” [32]. The review evaluated one RCT, one prospective comparative study and 10 case series across four different non-surgical interventions. Several studies have been published on MN interventions since 2015, but no subsequent systematic review assessing the quality of these studies or the effectiveness of the interventions has been published. An up-to-date synthesis of the available evidence is needed to assist clinicians in selecting the most effective treatments when managing patients with MN. The aim of this systematic review is to appraise and synthesise the evidence from a wide range of study designs investigating non-surgical interventions for MN.

## Methods

### Clinical question

To identify studies relating to non-surgical treatment of MN, a clinical question was defined using the population, intervention, comparison, outcome and study type (PICOS) format prior to establishing the search strategy. Population was defined as adults aged 18 years or older with a MN diagnosed through clinical symptoms, ultrasound or magnetic resonance imaging, without a history of significant trauma, foot surgery or systemic inflammatory conditions such as rheumatoid arthritis. Intervention was defined as any non-surgical intervention that aimed to reduce pain associated with MN. Surgery was defined as an incision into the body [33], thus non-surgical would include skin penetration interventions not involving an incision such as an injection or skin penetrating probe. The comparison was considered to be a control, or placebo group, or another non-surgical intervention compared to the primary intervention. Primary outcomes included pain or function, and secondary outcomes included intervention adverse events, change in neuroma size and quality of life. Included study types were experimental studies from level II (randomised controlled trials) to IV (case series with either post-test or pre-test/post-test outcomes) of the Australian National Health and Medical Research Council (NHMRC) Hierarchy of Evidence [34].

### Registration and reporting

The systematic review protocol was registered with the International Prospective Register of Systematic Reviews (PROSPERO) on 07 April 2016, registration no. CRD42016037405. The study has been reported according to the Preferred Reporting Items for Systematic Reviews and Meta-Analyses (PRISMA) recommendations to ensure transparent and complete reporting [35]. The types of studies to be included when the protocol was registered were the NHMRC study levels II through to III-3 [34], but the review group decided to include level IV studies due to the paucity of higher level evidence identified in initial searches.

### Information sources and search

The search was conducted up to 15 January 2018 for the following biomedical databases: CINAHL, EMBASE, Medline and Cochrane Central Register of Controlled Trials. A filter for human and English language studies was applied, without date restriction. The complete search strategy for Medline is shown in Table 1. Hand searching of reference lists of included studies was also conducted.

**Table 1:** Electronic search strategy for MEDLINE database, January 2018

#	Searches
1	peripheral nervous system neoplasms/ OR nerve compression syndromes/ OR nerve sheath neoplasms/ OR neuralgia/ OR neuritis/ OR (neuralgia$1 OR neuritis OR entrapment$1 OR (nerve ADJ5 compression)).ti,ab.
2	neurilemmoma/ OR neuroma/ OR neurofibroma/ OR (neurilemmoma$1 OR neuroma$1 OR neurofibroma$1).ti,ab.
3	foot diseases/ OR foot/ OR forefoot/ OR (foot OR forefoot).ti,ab.
4	metatarsus/ OR metatarsal bones/ OR metatarsophalangeal joint/ OR toes/ OR (metatarsus OR metatarsal$1 OR intermetatarsal OR metatarsophalangeal OR toe$1 OR interdigital OR (plantar ADJ5 digital)).ti,ab.
5	(morton$1 ADJ5 (disease$1 OR neuroma$1 OR neuralgia$1)).ti,ab. OR metatarsalgia/ OR metatarsalgia$1.ti,ab.
6	1 OR 2
7	3 OR 4
8	6 AND 7
9	5 OR 8
10	exp animals/ NOT humans.sh.
11	9 NOT 10
12	(treat$5 OR intervention$2 OR therap$5 OR manag$5 OR procedur$2).ti,ab.
13	11 AND 12 (apply English language filter)

### Study selection

**Fig. 1:**
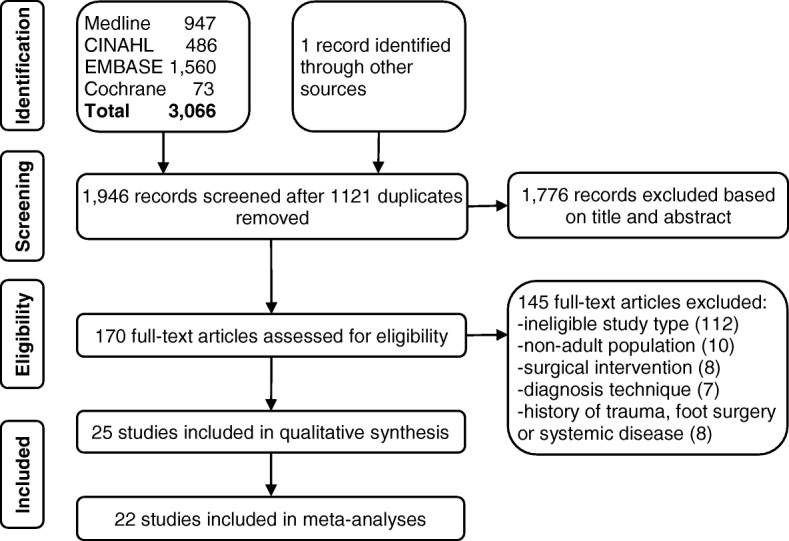
Flow diagram of study selection

All records retrieved by the search were independently screened by title and abstract (BM and MH) for relevance to the review question, then all potentially relevant studies underwent full text screening (BM, RH and MH). The PICOS clinical question described above formed the inclusion criteria by which detailed full-text evaluation was performed to determine which studies would be included in the review. Disagreements between the independent reviewers during study selection were resolved by a fourth reviewer (SH). The recommended PRISMA flow summary diagram [35] for the search strategy is shown in Fig. 1.

### Data collection process

A data collection form was developed and one reviewer (BM) extracted the data while a second reviewer (RH) checked the accuracy of the extracted data. A third reviewer (RW) provided clarification over data formats or data conversion. Data were extracted from each included study on (1) characteristics of study participants (including number of study participants, sex, age); (2) type of intervention (including type, technique, duration and frequency); (3) study methods (neuroma diagnosis method, type of outcome measure, length of follow up); and (4) study design (using the NHMRC Hierarchy of Evidence [34]). A study was defined as experimental if there were two parallel study groups, with allocation to group decided on either a randomised or quasi-randomised basis. A study was defined as an uncontrolled case series if there were at least two measurements (one pre-treatment and one post-treatment) in the same treatment group, with no concurrent comparison group. Numerical data were extracted, including number of participants in each group and frequency of successful outcomes for categorical data or mean and standard deviation for continuous data.

### Risk of bias in individual studies

All included studies were independently reviewed in full text for methodological quality (BM and MH) using the 27-item Downs and Black Quality Index [36]. Rating disagreements between the independent reviewers during study selection were resolved by a third reviewer (SH). The Quality Index provides a profile of each study’s methodological strengths and weaknesses. It has good test-retest (r = 0.88) and inter-rater (r = 0.75) reliability and can be used in both experimental and observational studies [36]. The Quality Index has previously been used to report the score with a Quality Index range of low, moderate and high [37].

For this systematic review all 27 items in the original Quality Index were used. Items one to four and six to 26 were given a maximum score of one point, item five a score of two points, and item 27 was scored on a 0–5 scale, to give a maximum possible score of 32. Item 27 was scored by calculating the *post-hoc* power of the study based on defining a minimal important difference of ten points on a 0–100 scale, extracting the standard deviation observed in the study, and specifying alpha = 0.05. Quality Index points associated with *post-hoc* study powers were zero (< 60% power), one (60 to < 80% power), two (80 to < 90% power), three (90 to < 95% power), four (95 to < 99% power), five (> 99% power) [38]. While no published data investigating the minimal important difference for MN was found, Thomson et al. [39], used a ten point change, Mahadevan et al. [40], a 15 point and Lizano-Diez et al. [41], a 30 point change on a 0–100 pain visual analogue scale (VAS). A VAS is a straight line, the ends of which are defined as the extreme limits of the sensation to be measured such as pain [42].

### Summary measures and synthesis of results

Raw results were extracted and when required, data were dichotomised into treatment success/failure. Interventions were categorised into groups of similar interventions, i.e. interventions with similar therapeutic targets or mechanisms (e.g. same class of drug used). Outcome measures were considered appropriate for synthesis if measuring the same construct (e.g. pain) and having a scale that could be combined for analysis (e.g. both continuous scales). The primary measure of treatment effect for binary data were odds ratio (OR) (experimental studies) or success rate (SR) (case series). The primary measure of treatment effect for continuous data were mean difference (MD) (for both experimental studies and case series). When estimating the MD for pre/post scores a correlation of 0.5 between pre and post measurements was assumed. When results from two or more studies are combined in a meta-analysis, overall estimates of effect are presented as weighted odds ratio, weighted success rate (WSR), or weighted mean difference (WMD) as appropriate. The precision of effect estimates was characterised using 95% confidence intervals (CI). Random effects models were used to pool treatment effects, and heterogeneity of clinical and methodological diversity was assessed with the *I*^*2*^ statistic [43]. Analyses were carried out separately for experimental studies and case series. For experimental studies with parallel groups, the between-group post-treatment scores were compared. For studies with pre/post data, if data were extracted from a case series, the within-group pre/post scores were compared, and if data were extracted from an experimental study with parallel-groups then the pre/post scores from the intervention arm were compared. The data for the RCT meta-analysis only includes RCT data and the pre/post case series meta-analyses only includes pre/post case series data.

**Fig. 2:**
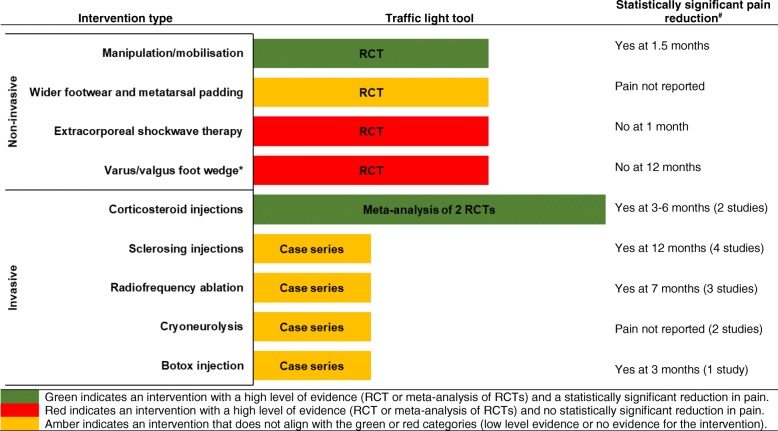
Clinical evidence summary: Morton’s neuroma non-surgical interventions for pain reduction. *RCT* Randomised controlled trial; Clinical evidence summary may be used in combination with the existing published treatment pathways for Morton’s neuroma; *No studies assessing the effect of orthoses on foot function related to Morton’s neuroma were found by the review; ^#^Statistically significant reduction in pain may not be a clinically significant reduction in pain (no data on the minimal important difference for pain reduction in Morton’s neuroma was found)

A clinical evidence summary (Fig. 2) was created using the traffic light tool to clearly display the key findings from the review. The traffic light tool has been rated as significantly more useful than the United Kingdom National Health Service recommended “situation, background, assessment, recommendation” system for communication within medical teams [44]. The traffic light tool has previously been used as a communication tool with health professionals for the management of foot care in diabetes [45] and the paediatric flat foot [46]. The figure and traffic light tool categorise interventions into three groups; (i) green to indicate an intervention with a high level of evidence (RCT or meta-analysis of RCTs) and a statistically significant reduction in pain, (ii) red to indicate an intervention with a high level of evidence (RCT or meta-analysis of RCTs) and no statistically significant reduction in pain, or (iii) amber for all interventions that don’t align with the green or red categories. The traffic light tool may be used in combination with the existing published treatment pathways for MN [25–30] when considering a non-surgical intervention. Study quality, effect size, adverse events (benefit versus harm), clinician experience, and the predicaments, rights, and preferences of the individual with MN will need to be balanced by the clinician in their decision making process [47].

## Results

### Overview of studies

The search identified 1946 potential titles and abstracts to screen after the removal of 1121 duplicates. Of the 170 studies requiring full text review, 25 were included [1–10, 19, 20, 22, 23, 39–41, 48–55]. The most common reason for excluding studies following full-text review was ineligible study type (112 studies). A flow diagram of the study selection process is presented in Fig. 1. Included studies encompassed a total of 1974 study participants and 2100 neuromas. Studies took place in United Kingdom (6 studies) [2, 7, 22, 39, 40, 50], Italy (5 studies) [8, 20, 23, 53, 55], United States of America (4 studies) [4, 5, 19, 49], Australia [1, 10], South Korea [52, 54], Spain [6, 41] and Turkey [3, 9] (2 studies each) and South Africa [48] and France [51] (1 study each). Clinical settings represented were Hospital (9 studies) [3, 8, 9, 22, 39, 41, 50, 52, 54], University [2, 6, 19, 20, 23, 48, 56] and Radiology [1, 7, 10, 49, 51, 53, 55] (7 studies each), and Private Practice (2 studies) [4, 5]. The study types consisted of seven RCTs [3, 39–41, 48, 50, 52], and 18 pre/post case series [1, 2, 4–10, 19, 20, 22, 23, 49, 51, 53–55].

### Outcome measures

Pain was measured by VAS in 18 studies [2, 6–10, 19, 23, 39–41, 48, 50–55]. All 0–10 point VAS were scaled to 0–100 point for data synthesis. Alternative categorical outcome measures included the Johnson scale [57] in nine studies [1, 7, 20, 22, 40, 41, 51, 52, 54] or overall satisfaction, improvement, treatment response or symptom relief. For the purposes of analysis, a treatment measured using the Johnson scale was classified as a success if the participant recorded “completely satisfied” and as a failure otherwise. Raw results are presented fully (Additional file 1). Outcomes were dichotomised as success/failure for meta-analysis. The mean time between treatment initiation and outcome recording was 9.9 months (3 weeks to 56 months).

### Types of intervention

**Fig. 3:**
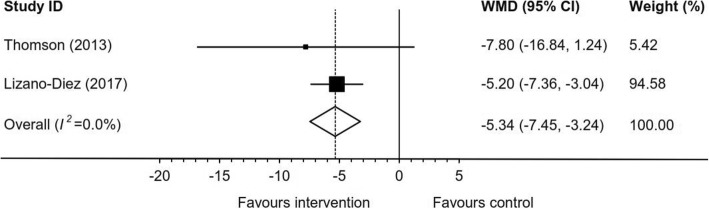
Meta-analysis for RCTs of corticosteroid injection on continuous outcomes (Pain VAS 0–100). *VAS* visual analogue scale; *WMD* weighted mean difference; *CI* confidence interval

**Fig. 4:**
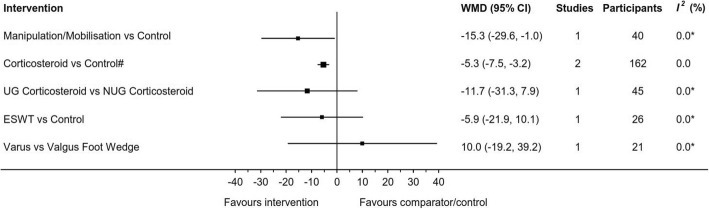
Mean difference (with 95% CI) for RCT continuous outcomes (Pain VAS 0–100). *Two or more studies required to calculate *I*^*2*^ statistic; #UG single injection and NUG three injections outcome data combined; *VAS* visual analogue scale; *MD* mean difference; *CI* confidence interval; *UG* ultrasound guided; *NUG* non-ultrasound guided; *ESWT* extracorporeal shock wave therapy

**Fig. 5:**
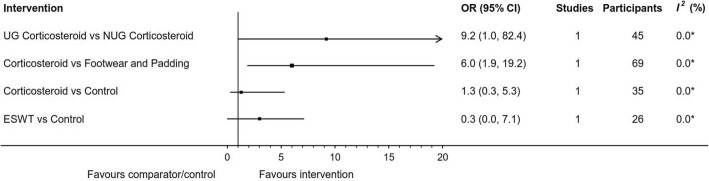
Odds ratio (with 95% CI) for RCT binary outcomes. *Three or more studies required to calculate *I*^*2*^ statistic; *OR* odds ratio; *CI* confidence interval; *UG* ultrasound guided; *NUG* non-ultrasound guided; *ESWT* Extracorporeal shockwave therapy

**Fig. 6:**
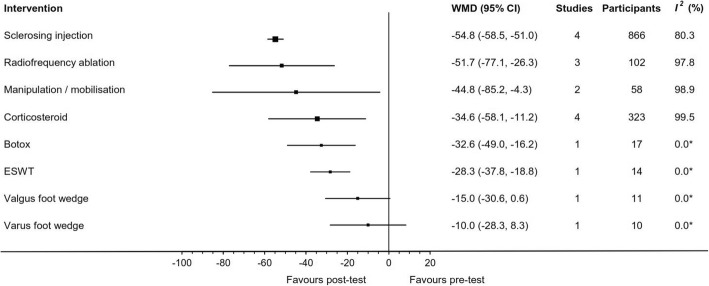
Effect of intervention on pre/post case series continuous outcomes (Pain VAS 0–100). *Two or more studies required to calculate *I*^*2*^ statistic; *VAS* visual analogue scale; *MD* mean difference; *CI* confidence interval; *ESWT* extracorporeal shock wave therapy

**Fig. 7:**
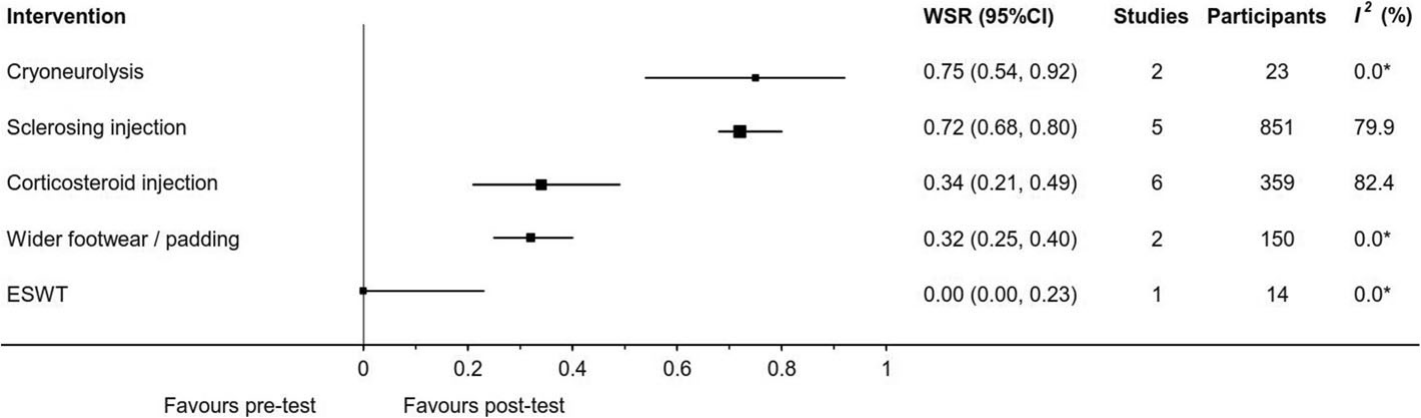
Effect of intervention on case series binary outcomes. *Three or more studies required to calculate *I*^*2*^ statistic; *SR* success rate; *CI* confidence interval; *ESWT* Extracorporeal shockwave therapy

The non-surgical interventions identified were divided into two categories: non-invasive (no skin penetration) and invasive. The non-invasive interventions included two mobilisation and manipulation studies [2, 48], two wider footwear and metatarsal padding studies [3, 4], one extracorporeal shockwave therapy (ESWT) study [52] and one orthoses study [50]. The invasive interventions included seven corticosteroid injection studies [1, 3, 22, 39–41, 54], seven sclerosing injection studies [5, 7, 8, 19, 20, 23, 55], three radiofrequency ablation studies [9, 10, 53], two cryoneurolysis studies [49, 51], and one Botox injection study [6]. See Table 2 for the study characteristics and Table 3 for the results of included studies. Overall recommendations arising from the meta-analysis using the traffic light tool are displayed in Fig. 2. Where studies were combined in meta-analysis, high levels of heterogeneity were generally observed, particularly for meta-analysis of pre/post studies; *I*^*2*^ values for meta-analyses are displayed in Figs. 3, 4, 5, 6 and 7.

**Table 2:** Characteristics of included studies

Study ID	Sample Size Female/Male	Age (years) Mean, Range	Inclusion Criteria	Intervention	Study Duration	Outcome Measure
Bennett 1995 [4]	115, 99/16	48.0, 18–79	Clinical	Wider, properly fitted footwear and a metatarsal pad proximal to the inflamed nerve	3 months	Satisfaction
Cashley 2015 [2]	38, 23/15	NR	Mulder’s sign, and positive digital nerve stretch test	Manipulation of the MTPJ (Distraction and plantarflexion of the MTPJ) 1/week × 4 weeks and 1 per 2 weeks × 2 (total = 6 treatments)	8 weeks	VAS (0–100) Pain pressure threshold
Cazzato 2016 [51]	20, 15/5	50.3, 24–67	Symptoms and US and MRI	1 x MRI guided cryoablation (0^0^ × 150 s) Some received additional cryoablation for 90 s but details NR	Mean 19.7 (1–50) months	VAS (0–10) Johnson scale
Chuter 2013 [10]	25, 21/4	55.0, 33–73	US	Mean 1.6 (1–3) x US guided radiofrequency ablation (81^0^, 5 × 2 min) 4 weeks apart	6 months	VAS (0–10)
Climent 2013 [6]	17, 7/10	58.2, SD 2.6	MRI or US	1 x Botox injection (50 units of onabotulinumtoxinA dissolved in 0.5 ml of normal saline)	3 months	VAS (0–10) FHSQ (foot pain, foot function, footwear, foot health)
Deniz 2015 [9]	20, 16/4	48.4, 21–67	Clinical and US	1 x US guided pulsed radiofrequency ablation (42^0^ × 5 min)	Mean 9 (7–15) months	Satisfaction Comfort when walking VAS (0–10)
Dockery 1999 [5]	100, 73/27	51.0, 20–75	Clinical but not specified	3–7 x alcohol and anaesthetic injection (0.5 ml of 4% sclerosing solution consisting of 48 ml of 0.5% bupivacaine HCI with epinephrine (1:200,000) and 2 ml of ethyl alcohol 98%) repeated at 5–10 day intervals	Mean 13 (6–24) months	Improvement
Fanucci 2003 [20]	40, 33/7	48.0, 28–65	Clinical and US	4 x US guided alcohol and anaesthetic injections (0.35 ml carbocaine and 0.15 ml ethylic alcohol 95%) 15 days apart	10 months	Johnson scale
Friedman 2012 [49]	5, 5/0	55.0, 47–60	US	1 x US guided cryoneurolysis (− 75^0^, 1–3 freeze/thaw × 2–3 min cycles)	Mean 16 (4–56) months	Treatment response
Govender 2007 [48]	20, NR, VS 20, NR Split for combined 10:3	50.0, NR, VS 53.0, NR Range for combined 23–79	Symptoms or clinical findings	Manipulation and mobilisation of foot and ankle joints (6 different techniques which may include distraction and plantarflexion of the MTPJ) AND 5 min detuned US 2/week × 3 weeks (total = 6 treatments) VS 5 min detuned US	3 weeks	VAS (0–100) Short-form McGill Pain Questionnaire Foot Function Index - Pain Foot Function Index - Disability Pain pressure threshold Pain pressure tolerance
Hassouna 2007 [22]	39, 32/7	55.8, 26–83	Clinical and US	1 x US guided steroid and anaesthetic injection (mixture of 2 ml 0.5% bupivacaine and 20 mg of triamcinolone acetonide)	Mean 11.4 (range NR) months	Activity restriction Footwear requirements Johnson scale
Hughes 2007 [7]	101, 84/17	53.8, 30–74	Clinical and US	4 x US guided alcohol and anaesthetic injections (0.1 ml ethyl alcohol 100% and 0.4 ml bupivacaine 0.25%) 14 days apart 4 additional injections provided to unknown number of subjects	Mean 10.5 (7–19) months	Johnson scale – modified VAS (0–10)
Hyer 2005 [19]	6, 4/2	61.7, 50–81	Clinical	3–9 x alcohol and anaesthetic injection (0.5 ml of 4% sclerosing solution consisting of 48 ml of 0.5% bupivacaine HCI with epinephrine (1:200,000) and 2 ml of ethyl alcohol 98%) weekly injections	Mean 346, SD 50.3 days	VAS (0–10)
Kilmartin 1994 [50]	10, NR, VS 11, NR Combined 19/2	40.0, SD 12.0 VS 46.0, SD 10.5	Clinical	Varus foot wedge made of 7 mm hard compressed felt on the under surface of a fibreboard inner to supinate the rearfoot and fitted into the subject’s shoes (low heeled lace-up or loose fitting slip on shoes). Felt was replaced at 4, 8 and 12 weeks. VS Valgus wedge to pronate the rearfoot	12 months	VAS (0–10)
Lizano-Diez 2017 [41]	16, 12/4 VS 19, 17/2	57.7, SD 9.8 VS 60.7, SD 11.6	Clinical, Mulder’s sign and MRI	1 x corticosteroid and anaesthetic injection (1 ml triamcinolone 40 mg and 1 ml mepivacaine 2%) 1/week for 3 weeks VS 1 x anaesthetic injection (2 ml mepivacaine 2%) 1/week for 3 weeks	6 months	VAS (0–100) AOFAS Johnson scale - modified
Magnan 2005 [23]	65, 55/10	51.5, 19–80	Clinical	1 or 2 electrostimulation guided alcohol and anaesthetic injections (2.5 ml phenol 5% water solution followed by 3 ml carbocaine 2%) (19 ft received 2nd injection within 3 months)	Mean 36 (24–55) months	VAS (effective / ineffective)
Mahadevan 2016 [40]	23, NR VS 22, NR	57.1, SD 11.7 VS 58.6 SD 14.3	Clinical and positive web space squeeze test and US	1 x US guided corticosteroid and anaesthetic injection (1 ml triamcinolone acetonide 40 mg and 2 ml lignocaine 1%) VS 1 x corticosteroid and anaesthetic injection (1 ml triamcinolone acetonide 40 mg and 2 ml lignocaine 1%)	12 months	VAS (0–100) Manchester-Oxford Foot Questionnaire Index Johnson scale
Markovic 2008 [1]	35, 28/7	54.0, 29–77	Symptoms and US	1 x US guided corticosteroid and anaesthetic injection (1 ml celestone chronodose 5.7 mg and 0.5 ml lidocaine 1%)	9 months	Johnson scale Lower extremity functional scale
Masala 2018 [53]	52, 46/6	53.0, 33–75	Symptoms and US and MRI	1 x US guided continuous radiofrequency ablation (85^0^ × 90 s)	12 months	VAS (0–10) FHSQ
Park 2017 [54]	201, 158/43	55.9, 23–80	Symptoms and Mulder’s sign and US	1 x US guided corticosteroid and anaesthetic (1 ml dexamethasone 5 mg and 1 ml lidocaine 1%)	6 months	VAS (0–10) Johnson scale
Pasquali 2015 [8]	508, 464/44	57.0, 29–81	Symptoms, clinical and US	Mean 3 (1–4) x US guided alcohol injection (0.3 ml ethyl alcohol 95% and 0.3 ml of mepivacaine 2%) 2 weeks apart then 4 weeks apart for 3rd and 4th injections	12 months	Satisfaction VAS (0–10)
Perini 2016 [55]	220, 187/33	55.8, 19–82	Clinical and US	1 x US guided alcohol and anaesthetic injection (0.6–1.0 ml of 50% absolute alcohol and 50% lidocaine 2%) every 2nd week for a median of 3 treatments (IQR 3–3)	Mean 19 (15–24) months	VAS (0–10)
Saygi 2005 [3]	35, 31/4 VS 34, 29/5	52.0, SD 11.8 VS 51.9, SD 11.0	Clinical and US	Shoes with wide toe boxes and low heels and metatarsal pad (custom fitted inserts) VS 2 x corticosteroid and anaesthetic injection (1 ml methylprednisolone acetate 40 mg and 1 ml prilocaine 4%) 3 weeks apart	6 months	Satisfaction
Seok 2016 [52]	14, 9/5 VS 12, 8/4	58.5, SD 13.4 VS 54.5, SD 14.3	Symptoms, Mulder’s sign and US	1 x ESWT (1000 shocks at 3 Hz) with energy level set to maximum patient tolerance (0.12–0.24 mJ/mm^2^) VS 1 x ESWT with probe perpendicular to energy delivery and energy level set to 0.03mJ/mm^2^	4 weeks	VAS (0–100) AOFAS Johnson scale
Thomson 2013 [39]	64, 54/10 VS 67, 57/10	54.7, SD 17.4 VS 51.6, SD 12.9	Symptoms and US	1 x US guided corticosteroid and anaesthetic injection (1 ml methylprednisolone 40 mg and 1 ml lignocaine 2%) VS 1 x US guided anaesthetic injection (1 ml lignocaine 2%)	3 months	Foot health thermometer VAS (0–100) MFPDI – pain MFPDI – walking MFPDI – work

*NR* Not reported, *MTPJ* Metatarsophalangeal joint, *VAS* Visual analogue scale, *US* Ultrasound, *MRI* Magnetic resonance imaging, *s* Second, *min* Minute, *SD* Standard deviation, *FHSQ* Foot health status questionnaire, *ml* Millilitres, VS Verses, *mg* Microgram, *mm* Millimetre, *IQR* Interquartile range, *ESWT* Extracorporeal shockwave therapy, *Hz* Hertz, *mJ* millijoules, *AOFAS* American Orthopaedic Foot and Ankle Score, *MFPDI* Manchester Foot Pain and Disability Index

**Table 3:** Results of included studies (listed by intervention type, NHMRC study level and Quality Index)

Study ID	NHMRC	Quality Index	Adverse Events (participants, %)	VAS MD (95% CI)	SR (95% CI)
Corticosteroid injection					
Thomson 2013 [39]	RCT	High	Corticosteroid group Several, (unknown %) increased foot pain 1–2 days post injection 3, (5.0%) dorsal skin hypopigmentation over injection site at 3 months 2, (3.0%) plantar fat pad atrophy at 3 months	− 23.9 (− 30.4 to − 17.4)	NR
Lizano-Diez 2017 [41]	RCT	Moderate	3, (18.7%) mild skin atrophy at the area of infiltration	−20.1 (− 21.8 to − 18.4)	38% (15 to 65)
Mahadevan 2016 [40]	RCT	Moderate	1, (2.2%) localised depigmentation	Ultrasound −40.8 (− 55.4 to − 26.2) Non-Ultrasound − 34.1 (− 48.7 to − 19.5)	Ultrasound 30% (13 to 53) Non-Ultrasound 5% (0 to 23)
Saygi 2005 [3]	RCT	Low	NR	NR	50% (32 to 68)
Park 2017 [54]	Case series	Moderate	NR	−57.0 (−59.4 to − 54.6)	20% (15 to 27)
Hassouna 2007 [22]	Case series	Low	NR	NR	31% (17 to 48)
Markovic 2008 [1]	Case series	Low	NR	NR	63% (41 to 81)
Sclerosing injection					
Perini 2016 [55]	Case series	Moderate	Common, (unknown %) short term pain at injection site 178, (80.9%) hypo anaesthesia of the innervated area	−60.0 (− 65.7 to − 54.3)	NR
Dockery 1999 [5]	Case series	Low	Common, (unknown %) post-injection neuritis for 48 h	NR	82% (73 to 89)
Fanucci 2003 [20]	Case series	Low	6, (15.0%) transitory plantar pain	NR	53% (36 to 68)
Hughes 2007 [7]	Case series	Low	Common, (unknown %) 5–10 s of moderate transient discomfort during injection 17, (16.8%) increased plantar pain 2 to 21 days 1, (1.0%) complex regional pain syndrome which resolved	− 55.0 (− 56.7 to − 53.2)	62% (52 to 72)
Hyer 2005 [19]	Case series	Low	NR	−61.2 (− 80.2 to − 42.2)	NR
Magnan 2005 [23]	Case series	Low	1, (1.8%) developed 4th toe pain but unsure if related to intervention	NR	80% (69 to 89)
Pasquali 2015 [8]	Case series	Low	Mean local inflammatory reaction 1-week post procedure 0.7, (range, 0 to 2)**	−51.0 (−52.9 to − 49.1)	74% (71 to 78)
Radiofrequency ablation					
Masala 2018 [53]	Case series	Moderate	NR	−73.0 (−74.7 to − 71.3)	NR
Chuter 2013 [10]	Case series	Low	8, (32.0%) described intervention as unpleasant 1, (4.0%) posterior tibial nerve irritation for 3 weeks	− 43.0 (− 50.8 to − 35.2)	NR
Deniz 2015 [9]	Case series	Low	2, (10.0%) superficial cellulitis and moderate haematoma	−38.0 (− 48.5 to − 27.5)	60% (36 to 81)
Manipulation/mobilisation					
Govender 2007 [48]	RCT	Moderate	NR	−24.0 (−31.3 to − 16.7)	NR
Cashley 2015 [2]	Case series	Low	NR	−65.3 (− 69.8 to − 60.8)	NR
Wider footwear and metatarsal padding					
Saygi 2005 [3]	RCT	Low	NR	NR	14% (5 to 30)
Bennett 1995 [4]	Case series	Low	NR	NR	38% (29 to 48)
Cryoneurolysis					
Cazzato 2016 [51]	Case series	Low	1, (4.2%) local cellulitis around cryo-probe entry point	NR	78% (52 to 94)
Friedman 2012 [49]	Case series	Low	NR	NR	60% (15 to 95)
Extracorporeal shockwave therapy					
Seok 2016 [52]	RCT	Moderate	NR	−28.3 (−37.8 to − 18.8)	0% (0 to 22)
Orthoses					
Kilmartin 1994 [50]	RCT	Moderate	1, (10%) lower limb pain with supination orthoses 2, (18.2%) lower limb pain with pronation orthoses	Supination −10.0 (− 28.3 to 8.3) Pronation − 15.0 (− 30.6 to 0.6)	NR
Botox injection					
Climent 2013 [6]	Case series	Low	NR	−32.6 (− 49.0 to − 16.2)	NR

*NHMRC* National Health and Medical Research Council levels of evidence [34]; *VAS* Visual analogue scale, *MD* Mean difference, *SR* Success rate, *CI* Confidence interval, *NR* None reported; ** 0 (no reaction), 1 (minimal swelling, pain, redness), or 2 (significant swelling, pain, redness)

### Quality of included studies

The quality assessment of individual studies can be viewed in Table 4. One study was rated as high, eight as moderate and 16 as low using the Quality Index, demonstrating a paucity of high-quality studies for the review to assess. Fifteen case series and one RCT study were rated as low. All studies were representative in terms of the staff and facilities where the public would normally receive the included interventions (Item 13). One study [10] did not report the main outcomes (Item 2) and another study [49] did not report participant characteristics (Item 3). Only four studies [39–41, 52] attempted to blind those measuring intervention outcomes (Item 15) and none concealed randomisation from staff until recruitment was complete (Item 24). Only Thomson et al. [39], achieved an adequate level of power allowing generalisability of results (Item 27).

**Table 4:** Quality index

Quality Index Items	Bennett 1995	Cashley 2015	Cazzato 2016	Chuter 2013	Climent 2013	Deniz 2015	Dockery 1999	Fanucci 2003	Friedman 2012	Govender 2007	Hassouna 2007	Hughes 2007	Hyer 2005	Kilmartin 1994	Lizano-Diez 2017	Magnan 2005	Mahadevan 2016	Markovic 2008	Masala 2018	Park 2017	Pasquali 2015	Perini 2016	Saygi 2005	Seok 2016	Thomson 2013
Reporting																									
1. Study hypothesis/aim/objective	1	1	1	1	1	1	1	1	0	1	1	1	1	1	1	0	1	1	0	1	1	1	1	1	1
2. Main outcomes	1	1	1	0	1	1	1	1	1	1	1	1	1	1	1	1	1	1	1	1	1	1	1	1	1
3. Participant characteristics	1	1	1	1	1	1	1	1	0	1	1	1	1	1	1	1	1	1	1	1	1	1	1	1	1
4. Interventions of interest	0	1	1	1	0	1	1	1	1	1	1	1	1	1	1	1	1	1	1	1	1	1	0	1	1
5. Distributions of principal confounders in each group	0	0	2	0	0	0	0	0	0	1	0	0	0	1	2	0	1	0	2	2	0	2	1	2	2
6. Main findings	1	1	1	1	1	1	1	1	1	1	1	1	1	1	1	0	1	0	1	1	1	1	1	1	1
7. Estimates of random variability for main outcomes	0	1	0	0	1	0	0	0	0	1	0	1	0	1	1	0	1	0	1	0	0	1	0	1	1
8. All the important adverse events that may be a consequence of intervention	0	0	1	0	0	1	0	0	1	0	1	0	0	1	0	0	1	1	1	0	0	0	0	0	0
9. Characteristics of patients lost to follow-up	0	1	0	1	1	1	1	1	1	1	0	0	0	0	0	1	0	0	1	1	1	1	0	0	0
10. Actual probability values for main outcomes	0	1	0	1	1	1	0	0	0	1	1	1	0	0	1	0	1	0	0	1	1	1	0	0	1
External validity																									
11. Were subjects who were asked to participate representative of the entire population from which they were recruited?	1	1	1	0	0	0	1	0	1	0	0	1	1	1	1	1	1	0	0	1	0	1	0	0	1
12. Were those subjects who were prepared to participate representative of the entire population from which they were recruited?	0	0	1	0	0	0	1	1	1	0	0	1	0	0	0	1	0	0	1	0	0	1	0	0	0
13. Were the staff, places, and facilities where the patients were treated, representative of the treatment the majority of subjects received?	1	1	1	1	1	1	1	1	1	1	1	1	1	1	1	1	1	1	1	1	1	1	1	1	1
Internal validity (bias)																									
14. Was an attempt made to blind study subjects to the intervention they have received?	0	0	0	0	0	0	0	0	0	1	0	0	0	0	1	0	1	0	0	0	0	0	0	1	1
15. Was an attempt made to blind those measuring the main outcomes of the intervention?	0	0	0	0	0	0	0	0	0	0	0	0	0	0	1	0	1	0	0	0	0	0	0	1	1
16. If any of the results of the study were based on “data dredging”, was this made clear?	1	1	1	1	0	1	1	1	1	1	1	1	1	1	1	1	1	0	1	1	1	1	0	1	1
17. Do analyses adjust for different lengths of follow-up?	0	1	0	0	1	0	0	1	0	1	0	0	0	1	1	0	1	1	1	1	1	0	1	1	1
18. Were the statistical tests used to assess the main outcomes appropriate?	0	0	1	0	0	0	0	0	0	1	1	1	0	1	1	0	1	0	1	1	1	1	1	1	1
19. Was compliance with the intervention reliable?	0	1	1	0	1	1	1	1	1	0	1	1	1	1	1	0	1	1	1	1	0	1	0	1	0
20. Were the main outcome measures valid and reliable?	1	1	1	1	1	0	0	1	0	1	0	0	1	1	1	1	1	1	1	1	0	1	0	1	1
Internal validity (selection bias)																									
21. Were the patients in different intervention groups recruited from the same population?	1	1	1	1	1	1	1	1	1	1	1	1	1	1	1	1	1	1	1	1	1	1	0	1	1
22. Were study subjects in different intervention groups recruited over the same period of time?	1	1	1	1	1	1	1	1	1	1	1	1	1	1	1	1	1	1	1	1	1	1	0	0	1
23. Were study subjects randomised to intervention groups?	0	0	0	0	0	0	0	0	0	1	0	0	0	1	1	0	1	0	0	0	0	0	1	1	1
24. Was the randomised intervention assignment concealed from both patients and staff until recruitment was complete and irrevocable?	0	0	0	0	0	0	0	0	0	0	0	0	0	0	0	0	0	0	0	0	0	0	0	0	0
25. Was there adequate adjustment for confounding in the analyses from which the main findings were drawn?	0	0	0	0	0	0	0	0	0	0	0	0	0	1	0	0	0	0	0	0	0	0	1	0	1
26. Were losses of patients to follow-up taken into account?	1	1	0	1	1	1	1	1	1	1	0	1	0	1	0	1	1	0	1	1	1	1	0	0	1
Power																									
27. Did the study have sufficient power to detect a clinically important effect where the probability for a difference being due to chance is less than 5%?	0	0	0	0	0	0	0	0	0	0	0	0	0	0	0	0	0	0	0	0	0	0	0	0	1
Total score (/32)	11	17	17	12	14	14	14	15	13	19	13	16	12	20	21	12	22	11	19	19	14	20	10	18	23
Quality Index (low 0–17, moderate 18–22, high 23–32)	low	low	low	low	low	low	low	low	low	moderate	low	low	low	moderate	moderate	low	moderate	low	moderate	moderate	low	moderate	low	moderate	high

*Item 27* was scored by calculating the post-hoc power of the study based on defining a minimal important difference of ten points on a 0–100 scale, extracting the standard deviation observed in the study, and specifying alpha = 0.05. Checklist points associated with post-hoc study power < 60% = 0, 60 to < 80% = 1, 80 to < 90% = 2, 90 to < 95% = 3, 95 to < 99% = 4, > 99% = 5; *Quality Index* modified from Barton et al. [37]

### Non-invasive interventions

#### Manipulation/mobilisation

Manipulation/mobilisation (involving distraction and plantarflexion of the metatarsophalangeal joints and mobilisation of other foot and ankle joints as required) was reported in two studies [2, 48]. One study was an RCT [48] that showed an effect in favour of the treatment over control at 6 weeks (MD: -15.3, 95%CI: -29.6 to − 1.0) (Fig. 4) and the other was an uncontrolled pre/post study [2] (Table 3). The two studies showed a pre/post reduction in pain at the end of the sixth treatment, mean 5.5 weeks (range 3 to 8) (WMD: -44.8, 95%CI: -85.2 to − 4.3) (Fig. 6).

#### Wider footwear and metatarsal padding

Properly fitted footwear with a wide toe box, low heel and a metatarsal pad was assessed in two studies [3, 4]. An RCT [3] showed a statistically significant success rate where the odds of a non-ultrasound guided (NUG) corticosteroid injection and anaesthetic (intervention) being successful were 6 times greater than the odds of footwear and padding (control) being successful at 6 months (OR: 6.0, 95%CI: 1.9 to 19.2) (Fig. 5). The other was an uncontrolled pre/post study [4]. Combining these two studies showed footwear and padding to be successful in 32% of participants at a mean of 4.5 months (range 3 to 6) (WSR: 32, 95%CI: 25 to 40%) (Fig. 7).

#### Extracorporeal shockwave therapy

One study investigated ESWT, which involves microsonic energy (shockwave) pulses delivered to the plantar forefoot [52]. An RCT comparing ESWT to sham ESWT showed no statistically significant treatment effect for ESWT at 1 month review (OR: 0.3, 95%CI: 0 to 7.1) (Fig. 5), and no statistically significant reduction in pain (MD: -5.9, 95%CI: -21.9 to 10.1) (Fig. 4). Additional results are available in Figs. 6 and 7.

#### Varus/valgus foot wedge

The varus/valgus foot wedges used in the study were a cobra style hard compressed felt padding adhered to the plantar surface of a fibreboard insole to supinate or pronate the foot. These were worn in the participant’s usual footwear (low heeled lace-up or loose fitting slip on shoes) [50]. This RCT comparing the foot wedges showed no statistically significant treatment effect by the varus wedge over the valgus wedge at a 12 month review (MD: 10.0, 95%CI: -19.2 to 39.2) (Fig. 4). Additional results are available in Fig. 6.

### Invasive interventions

#### Corticosteroid injection

A corticosteroid and anaesthetic injection at the site of the neuroma was investigated by seven studies. Three studies were RCTs, one comparing ultrasound guided (UG) corticosteroid and anaesthetic injection to UG anaesthetic injection [39], a second comparing NUG corticosteroid injection to NUG anaesthetic injection [41] and a third study comparing UG to NUG corticosteroid injection [40]. The remaining three studies of UG corticosteroid injections were uncontrolled case series [1, 22, 54]. Meta-analysis of two trials [39, 41] comparing corticosteroid and anaesthetic injection combined against anaesthetic injection alone (mean follow-up time = 4.5 months; range 3 to 6) favoured the combined intervention (WMD: -5.3, 95%CI: -7.5 to − 3.2) (Fig. 3). Additional results from RCTs are displayed in Figs. 4 and 5. Continuous outcome measures for four studies [39–41, 54] showed a pre/post reduction in pain at a mean of 6.8 months (range 3 to 12) (WMD: -34.6, 95%CI: -58.1 to − 11.2) (Fig. 6). Binary outcome measures with six studies [1, 3, 22, 40, 41, 54] demonstrated success following intervention at a mean of 8.4 months (range 6 to 12) (WSR: 34, 95%CI: 21 to 49%) (Fig. 7).

#### Sclerosing injection

Seven studies evaluated sclerosing injections, which involved either an UG (4 studies) [7, 8, 20, 55] or NUG (3 studies) [5, 19, 23] injection of a sclerosing compound combined with local anaesthetic. Sclerosing compounds included ethyl alcohol (6 studies) [5, 7, 8, 19, 20, 55] or phenol (1 study) [23]. The number of injections ranged from one to nine over a period of up to 3 months. All seven studies were uncontrolled pre/post studies. Four of the seven studies [7, 8, 19, 55] using continuous outcome measures showed a pre/post reduction in pain after a mean 13.4 months (range 7 to 24) review period (WMD: -54.8, 95%CI: -58.5 to − 51.0) (Fig. 6). Five of the seven studies [5, 7, 8, 20, 23] with binary outcome measures showed success favoured sclerosing injections after a mean 16.3 months (range 6 to 55) review period (WSR: 72, 95%CI: 68 to 80%) (Fig. 7).

#### Radiofrequency ablation

Three studies [9, 10, 53] utilised radiofrequency ablation, where an UG probe is inserted into the neuroma and heated for a pulsed or continuous treatment. All studies had a pre/post design with no control group. The three studies showed treatment effects in favour of the intervention, (WMD: -51.7, 95%CI: -77.1 to − 26.3) after a mean review period of 7 months (range 6 to 15) (Fig. 6).

#### Cryoneurolysis

Two studies [49, 51] investigated cryoneurolysis, where an UG probe is inserted into the neuroma for one to three freeze/thaw treatment cycles. Both were uncontrolled pre/post studies. Two studies showed the success rate favoured treatment (WSR: 75, 95%CI: 54 to 92%) after a mean review period of 11.4 months (range 1 to 50) (Fig. 7).

#### Botox injection

One study evaluated a NUG single injection of 50 units of onabotulinumtoxinA dissolved in 0.5 ml of normal saline [6]. The study had an uncontrolled pre/post design with 17 participants showed treatment effects in favour of the intervention (WMD: -32.6, 95%CI: -49.0 to − 16.2) after a review period of 3 months (Fig. 6).

#### Change in neuroma size

Four studies [7, 10, 20, 52] used ultrasound to report on change in neuroma size. Ultrasound has been reported as a reliable modality to measure neuroma size [58]. Three case series [7, 10, 20] reported on pre/post intervention change in symptomatic neuroma size and one experimental study [52] reported on post change between groups. Fanucci et al. [20], reported a pre/post mass volume reduction of at least 20% in 21 of 40 participants with UG sclerosing injections. These 21 participants were the only participants to report “complete satisfaction” on the Johnson scale. Hughes et al. [7], found a pre/post reduction in diameter of 30% in 30 of 101 participants with UG sclerosing injections. Chuter et al. [10], was unable to compare pre/post change in diameter due to the much less distinct and ill-defined neuroma appearance after UG radiofrequency ablation. Seok et al. [52], comparing ESWT to control reported the mean diameter in both groups slightly reduced post intervention but no statistically significant difference was found.

#### Adverse events

No adverse events were reported for mobilisation and manipulation [2, 48], wider footwear and metatarsal padding [3, 4] or Botox injection interventions [6] (Table 3). Corticosteroid injections [39–41], sclerosing injections [5, 7, 8, 20, 23, 55], radiofrequency ablation [9, 10], cryoneurolysis [51] and orthoses [50] all reported adverse events. The most common event being pain during and post sclerosing injection [5, 7, 8, 20, 23, 55] and the most serious being dorsal skin hypopigmentation [39, 40], skin atrophy [41] and plantar fat pad atrophy [39] 3 months after a corticosteroid injection.

## Discussion

This systematic review evaluated non-surgical interventions for MN, including a wide range of study types to assist clinicians in their evidence-based management of MN and to inform the future direction of research. The review identified seven RCTs [3, 39–41, 48, 50, 52] and 18 case series [1, 2, 4–10, 19, 20, 22, 23, 49, 51, 53–55] with only one high quality RCT [39]. Meta-analysis of two RCTs [39, 41] found that corticosteroid injection decreased pain more than control. One RCT [3] showed that corticosteroid injection was superior to footwear and padding when measuring treatment success. Manipulation/mobilisation demonstrated some efficacy when compared to control at 6 weeks in another RCT [48]. Several case series found a reduction in pre/post pain with sclerosing injections [5, 7, 8, 19, 20, 23, 55], radiofrequency ablation [9, 10, 53], cryoneurolysis [49, 51], and Botox injection [6] but these results should be interpreted with caution. No statistically significant reduction in pain was demonstrated by an RCT [52] investigating ESWT compared to control or by another RCT [50] comparing varus/valgus foot wedges.

The meta-analysis of corticosteroid injections combined two RCTs [39, 41] which varied in follow-up (3 vs 6 months), number of injections (1 vs 3) and guidance during injection (UG vs NUG). Six of the studies [1, 3, 22, 40, 41, 54] had a 6 month or longer follow-up and only one study [39] had a 3 month follow-up (seven studies in total). It is not known whether combining a 3 month difference in follow-up periods affects the quality of the analysis. Five of the seven included studies used one injection [1, 22, 39, 54, 56], however two published treatment pathways for MN [26, 30] state that up to three injections are typically given. Thomas et al. [25], reports multiple injections obtain better results, however this statement is based on low quality studies [3, 4, 59–61]. There is no high-quality evidence for the number of injections or if multiple injections influence the effect size more than one injection. Mahadevan et al. [40], demonstrated there was no statistically significant difference between UG and NUG corticosteroid injections when a trained clinician administered the NUG injection (Figs. 4 and 5). This finding was used to justify combining the two RCTs [39, 41] for the meta-analysis. Neuroma diameter may be a factor in the small reduction in pain reported by the corticosteroid meta-analysis. Mahadevan et al. [56], found that people with neuroma with a transverse diameter larger than 5 mm had worse pain scores by 6 months post injection compared to those with a smaller neuroma. The mean transverse diameter for Thomson et al. [39], was 9.6 mm and Lizano-Diez et al. [41], was 8 mm which were both larger than 5 mm. The follow-up period and number of injections are differences which should be considered when interpreting the small effect size reported in the meta-analysis (Fig. 3).

One RCT of manipulation/mobilisation [48] had the largest mean difference of the included RCTs in the review (Fig. 4). The short follow-up period (1.5 months) compared to the follow-up mean of all the included studies in the review (9.9 months) suggests the result should be interpreted with caution. The intervention involved several manipulations/mobilisations including dorso/plantar glides of one metatarsal relative to the next and distraction and/or plantarflexion of the metatarsophalangeal joints. The common plantar digital nerve is surrounded by concentric layers of fibrous and loose connective tissue which creates a protective tunnel for the nerve to move independently of the surrounding tissue during gait [62]. A proposed pathological process involves the connective tissue becoming thickened and fibrotic in Morton’s neuroma [63] changing the protective tunnel into a nerve entrapment with ischemia [62]. Manipulation/mobilisation may reduce pain by decreasing the stiffness in the connective tissue surrounding the nerve.

The studies assessing footwear and padding [3, 4] are of low methodological quality demonstrating small success rates and one RCT [3] showed corticosteroid injections to be more successful than footwear and padding, however it should be considered that there are no adverse events reported with footwear and padding. A metatarsal pad is shaped to fit the plantar aspect of the foot, proximal to the metatarsal heads at the distal border, medially and laterally to the first and fourth intermetatarsal spaces respectively and proximal to the metatarsal bases with the pad thickness reducing distal to proximal and toward the medial and lateral borders. Prefabricated or custom foot orthoses may incorporate a metatarsal pad.

The included sclerosing injection case series [5, 7, 8, 19, 20, 23, 55] encompassed varying frequency of injections (1 to 9 injections over a period of up to 3 months) and review periods (6 to 55 months). While the WMD in pain scores and WSR were high (Figs. 6 and 7), the treatment and review variability coupled with only uncontrolled pre/post data provides low quality evidence and an RCT is required to confirm these findings. Three published treatment pathways include sclerosing injections as a second stage intervention [25, 26, 28], while another three report concern about the lack of high quality evidence [27], adverse events [29, 30] or the lack of long term treatment success leading to surgery [29, 30]. Pain during and post injection for up to 21 days was a common short-term adverse event, but no long-term adverse events were reported from 1040 participants with a mean 14.9 months (range 6 to 55) review period. Failure of the sclerosing injection to substantially reduce pain in either the short or long term resulted in 177 (17%) participants progressing to surgical excision.

Three pre/post case series investigating radiofrequency ablation [9, 10, 53] found a large reduction in pain (WMD: -51.7; 95%CI: -77.1 to − 26.3) (Fig. 6) but an RCT is required to confirm these findings. While radiofrequency ablations have been investigated previously, they have not been included in treatment pathways to date because participants in previously identified radiofrequency ablation studies concurrently received a corticosteroid injection, which made it difficult to separate the benefits of the two interventions [30]. The three new pre/post case series [9, 10, 53] included in this systematic review are not confounded by additional interventions and demonstrate pain reduction with no long-term adverse events reported.

Kilmartin and Wallace [50], assessed a varus felt wedge to supinate the foot verses a valgus felt wedge to pronate the foot, and reported no statistically significant reduction in pain with either type of wedge. The study tests the hypothesis that a supinated subtalar joint would reduce forefoot abduction, reducing MN compression and pain. This hypothesis, based on the Root model of foot function, assumes that pronation of the foot during the propulsive phase of gait causes the metatarsals to become unstable, resulting in the metatarsal heads moving laterally, causing a shear force on the plantar soft tissues, resulting in a MN [64]. The current understanding is that foot orthoses alter the magnitude, timing, and velocity of motion in the foot. In addition to altering kinematics, foot orthoses also alter plantar pressures, muscle activity and kinetics, which can be used to reduce stress on targeted tissues with the intention of reducing the risk of tissue damage [65–68]. The term orthosis used in the study [50] does not represent the current understanding of foot or orthosis function. Therefore, the term “varus/valgus foot wedge” has been used in this review to represent the intervention used in Kilmartin and Wallace’s study. No studies assessing the effect of orthoses on foot function related to MN were found by the review.

This systematic review provides the most current and comprehensive assessment of non-surgical interventions for MN published to the best of the authors’ knowledge. Clinicians and researchers may use the findings as an evidence-based summary to help guide their clinical management and research. The results however, should be interpreted while considering the following limitations. The review only searched for and included studies published in English, excluding non-English language publications. A 2009 RCT comparing ESWT to sham ESWT was not included in this review as the participants mean age and range was not reported [69]. Including this RCT may have altered the results from the data analysis. The Johnson scale [57] was collapsed into binary data, by classifying “satisfied” as success. Regrouping binary data with “satisfied” and “satisfied with minor reservations” as success would increase the success rates reported and modify the results. There is no published minimal important difference for MN interventions. Therefore, it is unknown whether the small effect sizes reported by RCTs are clinically meaningful. Where enough studies were pooled to calculate an *I*^*2*^ statistic with pre/post continuous (Fig. 6) or binary (Fig. 7) outcomes, the values ranged from 79.9 to 99.5%. Higgins et al. [43], showed about a quarter of the meta-analyses in the Cochrane database of systematic reviews have *I*^*2*^ values over 50%. Values over 75% have tentatively been assigned as having high levels of heterogeneity [43]. Where *I*^*2*^ values were calculated in this systematic review for pre/post data there were high levels of heterogeneity.

High quality experimental studies investigating non-surgical treatments for MN are lacking [30, 32, 39], although there has been a small number of RCTs recently published [40, 41, 52]. In particular, future RCTs are needed to evaluate the effectiveness of sclerosing injections, radiofrequency ablations and cryoneurolysis, in order to confirm findings from low quality case series. Using a randomised study design where an intervention group is compared with a control group allows researchers to measure the causal benefit of the intervention. That is, the effect of intervention beyond any placebo effect a sham treatment may have, or beyond any natural improvement with time, which cannot be determined in a pre/post case series. The identification of a minimal important difference for pain improvement [70] would be informative to help clinicians and researchers understand the minimum worthwhile improvement patients report, replacing the range of values demonstrated across three RCTs included in this review [39–41]. When designing experimental studies, consideration should be given to consistent review periods (1, 6 and 12 months), validated outcome measures and the CONSORT guidelines [71] to allow the combining of RCTs for high quality meta-analyses of interventions with the same therapeutic mechanism.

## Conclusions

This review found some evidence of pain reduction following corticosteroid injection or manipulation/mobilisation techniques for MN. However, no high-quality evidence currently exists to inform which intervention should be the gold standard for first- or second-line non-surgical treatments. Further high quality RCTs are warranted to provide a solid evidence base for non-surgical treatment of MN.

## Additional file

Additional file 1: Raw results for outcome measures of included studies. (XLSX 22 kb)

## Abbreviations

CI: Confidence interval
ESWT: Extracorporeal shockwave therapy
MD: Mean difference
MN: Morton’s neuroma
NHMRC: Australian National Health and Medical Research Council
NUG: Non-ultrasound guided
OR: Odds ratio
PICOS: Population, intervention, comparison, outcome and study type
PRISMA: Preferred reporting items for systematic reviews and meta-analyses
RCT: Randomised controlled trial
SR: Success rate
UG: Ultrasound guided
VAS: Visual analogue scale
WMD: Weighted mean difference
WSR: Weighted success rate
